# The Treatment of Hepato-Intestinal Toxæmia.—II

**Published:** 1909-06-05

**Authors:** 


### SPECIAL ARTICLES.

### THE TREATMENT OF HEPATO-INTESTINAL TOXÆMIA.—II.

The treatment advocated by Dr. Quintard for the hepato-intestinal toxæmia he describes is as follows: The first thing to decide is whether the patient should be put to bed or not. If the condition has existed for a long time, or if it is particularly severe; if the nervous symptoms are especially prevalent, particularly in the case of women; if anæmia has reached a severe degree; or if such conditions as myasthenia gastrica or concomitant gastro-intestinal inflammation exist: then these patients should be put to bed and kept there for from three weeks to two months. If possible this rest cure should be undertaken away from the patient's own home and immediate family, and no communication should be held between him and his friends until such time as the physician thinks right. Cases of hepato-intestinal toxæmia, complicated as they frequently are by neurasthenia, and possibly by visceral disorders, are difficult to treat, and unless for the time being their entire mode of life is under the strict surveillance of their medical adviser, such cases often drift from bad to worse, and in the end read disaster for doctor and patient alike. It is well at the onset to inform both the patient and his family that it will take weeks, and probably months, for the recovery to be complete.

Each case should be thoroughly examined at the outset; it may be a week before one can complete both the physical examinations, and also those of the blood, the urine, the gastric contents, and the fæces. During such period the patients may be up and about, but whenever practicable it is far better to have them in bed, preferably in a hospital or institution where such examinations can be carried out in a thorough and systematic manner. Failure to cure these toxic conditions may frequently be traced to lack of thorough investigation.

Diet is an all-important matter in case of hepato-intestinal toxæmia, but one cannot emphasise too fully the fact that there can be no definite or dogmatic rules given to cover all cases. The results of the physical and clinical examinations obtained in each individual case must in a great measure be the guide. Certain general principles can, however, be laid down. The use of tobacco and of alcoholic beverages should be positively prohibited. Coffee should be interdicted as a rule. Highly seasoned foods, condiments, salted, dried, smoked, or pickled foods of any kind should not be allowed. At times plain pudding can be given in moderation, but sugary sweets, chocolate, cakes, and so forth are dangerous. Sugar itself should be reduced to a minimum. Otherwise good, plain food in reasonable quantities and at regular times can be permitted.

When the patient is in bed, massage, Swedish movements, and perhaps hydrotherapy, are great aids to the treatment. Regular daily massage should always be given; but mildly at the start, and in those who are exceedingly nervous it may be wise to wait a week before beginning it. Above all, everything for these patients should be according to rule, time, and definite system. Schedules for each patient should be made out and handed to the nurse. Under no circumstances, however, should the patients be permitted to see either their charts or their schedules.

So far as drugs are concerned, the first object is to rid the system of accumulated poison. This is accomplished by flushing out the liver and the intestines. Apart from special treatment rendered necessary by definite gastro-intestinal complications, it is Dr. Quintard's rule to give these patients three times a day before each of the principal meals 10 grains of glycerophosphate of soda in a large glass of hot water, to be sipped slowly. This may be continued for many weeks. He also administers to these patients every other day for the first week a mercurial in the following form:

| | | |
|---|---|---|
| ℞ | Ext. nucis vomicæ ... ... | gr. $\frac{1}{8}$ |
| | Massæ hydrarg. . . ... | gr. v. |
| | Ext. colocynth. comp. ... | gr. iij. |
| | Ext. rhei ... ... ... | gr. vij. |

Misce et divide in capsulas duas.
Sig. : Both capsules to be taken at night.

This is to be followed the next morning by a dose of citrate of magnesia or Rochelle salts, or phosphate of soda, or one of its various preparations. On the morning when such saline is given that morning's dose of glycerophosphate of soda is omitted. The second week the above mercurial is given twice, and during the third week only once.

It may be well to remark particularly that blue mass is far more efficacious in these cases than is calomel. The latter at times acts too violently and leaves the patients depressed, with feelings of nausea and chilly sensations that may last as long as 48 hours after the dose has been taken. Moreover, after calomel one is more liable to have such symptoms as breathlessness on exertion than after the use of blue mass. The course of treatment with blue mass and salines may have to be repeated. If there is a particular desire to give calomel it should be given in quite small doses—gr. $\frac{1}{4}$ to $\frac{1}{2}$—either alone or with salol or carbonate of soda.

If there is any actual colitis or entero-colitis, or if constipation has existed for a long time, or if the toxæmia is severe and accompanied by intestinal flatulency, it is well to resort to high colonic lavage. The solutions commonly employed for this are either normal saline or else Carlsbad salts in water, a drachm or two drachms to the quart. The water should be fairly hot to avoid abdominal cramps.

The anæmia will need attention. After the first week or ten days, when the liver and intestine have been flushed out to some extent, one may begin to give the blander preparations of iron. The pyrophosphate in one-grain doses in capsules or solution, or the peptomanganate, are good. No iron tonic should be given until the toxæmia is at least partly under control. Later, when an iron tonic treatment can be adopted with less reserve, the following prescription will be found serviceable:—

| | | |
|---|---|---|
| ℞ | Acidi arseniosi ... ... | gr. $\frac{1}{30}$ |
| | Strychninæ phosphatis ... | gr. $\frac{1}{40}$ |
| | Ferri pyrophosphatis ... | gr. j. |

Misce et mitte in formâ capsulæ.
Sig. : Take one capsule three times a day after meals.

Or as an alternative:—

| | | |
|---|---|---|
| ℞ | Strychninæ glycerophosphatis | gr. $\frac{1}{40}$ |
| | Ferri glycerophosphatis ... | gr. j. |
| | Mangan. glycerophosphatis | gr. ij. |

Misce et mitte in formâ capsulæ.
Sig. : Take one capsule thrice daily after meals.

For the nervous manifestations in these cases, especially at the onset, there is nothing better than the combinations of bromides. Five grains each of ammonium, potassium, and sodium bromide given in plain water two or three times daily act better than any other sedative in these cases. When neurasthenia is a complication the preparations of valerian, especially valerianate of zinc in 1- or 2-grain doses three times a day, seem to act well. Strychnine and its various combinations may be given with advantage where the patients are in need of such a neuro-muscular stimulant and tonic, but if the patients are very nervous or if the toxæmia is severe strychnine should not be given at the start. The same may be said of arsenic and its various preparations.

